# Longitudinal analysis of bone metabolism using SPECT/CT and ^99m^Tc-diphosphono-propanedicarboxylic acid: comparison of visual and quantitative analysis

**DOI:** 10.1186/s13550-016-0217-4

**Published:** 2016-07-28

**Authors:** Michael Beck, James C. Sanders, Philipp Ritt, Julia Reinfelder, Torsten Kuwert

**Affiliations:** 1 Clinic of Nuclear Medicine, University of Erlangen-Nuremberg, Ulmenweg 18, 91054 Erlangen, Germany; 2 Pattern Recognition Lab, University of Erlangen-Nuremberg, Erlangen, Germany

**Keywords:** Bone scintigraphy, SPECT/CT, Absolute quantification, SUV

## Abstract

**Background:**

The therapy response of osseous metastases (OM) is commonly monitored by bone scintigraphies (BS). The aim of this study was to compare visual evaluation of changes in tracer uptake with quantitation in absolute units in OMs; 52 OMs from 19 patients who underwent BS with SPECT/CT at time points one and two (TP1/2) were analyzed retrospectively, with an average of 10.3 months between TP1 and 2. Tracer uptake in lesions was visually compared by two independent readers in both planar scintigraphies and SPECT/CT across both TPs and classified as regressive, stable, or progressive. Quantitative analysis was performed by measuring peak standardized uptake values (SUV). Based on quantitation, lesions were similarly classified as regressive (>30 % decrease), progressive (>30 % increase), or stable (rest). If available, uptake in reference regions in the lower thoracic or lumbar spine was used for normalization.

**Results:**

In OMs at TP1 and TP2, mean SUV_peak_ (±SD) was found to be 20.4 (±20.8) and 16.4 (±11.5), respectively. For the reference region, mean SUV_mean_ was 5.6 (±1.9) and 4.9 (±2.2). Agreement between quantitative and visual assessment was only moderate, with an average Cohen’s kappa of 0.42 for planar scintigraphy and 0.62 for SPECT/CT. Discrepancies occurred in between 11 and 22 of the 52 lesions, depending on the reader and whether planar or SPECT imaging was considered.

**Conclusions:**

Compared to measuring uptake in absolute units, visual evaluation of skeletal scintigraphies for change in tumor metabolism yields inconsistent results in roughly one third of the cases.

## Background

Due to its high sensitivity, bone scintigraphy is part of the standard diagnostic workup in common cancer diseases such as prostate and breast cancer [1–4]. Commonly, regression or progression of bone metastases under therapy is determined by the decrease or increase, respectively, in the occurrence of lesions in consecutive bone scintigraphies. This is routinely performed by visual interpretation.

However, visual interpretation of planar scintigraphy and single photon emission computed tomography (SPECT)(/CT) is a subjective exercise and highly user-dependent. A standardized, semi-quantitative, more user-independent evaluation has not yet been established for the clinical routine.

Unfortunately, data on techniques for long-term bone scan evaluation and correlation with clinical disease progression is scarce, remaining a domain of magnetic resonance imaging (MRI) [5], where the size of lesions can be reliably tracked. Alternatively, positron emission tomography (PET)/CT is applied [6–8], where the direct measurement of tumor glycolysis and the ability to estimate standardized uptake values (SUV) allows an objective comparison of consecutive acquisitions [7, 9].

The superiority of combined SPECT/CT over conventional SPECT and planar scintigraphy in bone scans has been shown in various studies [10–12]. Modern iterative SPECT/CT reconstruction methods also allow for quantitative estimation of local tracer uptake in specified volumes in absolute units of kiloBecquerel per milliliter. They employ corrections for photon attenuation via data retrieved from CT, as well as for photon scatter and the point spread function [13–15], among other factors. Following system calibration and normalization for body weight/surface and injected dose, the calculation of SUVs in reconstructed SPECT/CT images from patient studies is feasible with an acceptable error [16]. Quantitative measurements carry the potential to overcome the disadvantages of a purely visual analysis. Reading conventional gamma camera images generally has two sources of variance. The first of these is the reproducibility of placement of the volume of interest (VOI) to be evaluated, and the second is the actual assessment of the local amount of tracer uptake. While not alleviating the need for manual VOI placement, quantitative measurements replace the potentially subjective determination of uptake in the VOI, thus minimizing the latter cause of variance, leading to increased intra- and inter-reader reproducibility.

The aim of this study was to assess the agreement between visual and quantitative evaluation of changes in local tracer uptake for patients with malignant bone lesions.

## Methods

### Patients

Our analysis was approved by the local ethics commission of the university Erlangen-Nuremberg medical faculty (application number 4053). Due to the retrospective nature of the analysis, written consent from the patients was not necessary. From our clinical database, we retrieved image datasets from patients with osseous metastases due to breast or prostate cancer who had undergone two planar bone scintigraphies and standard SPECT/CT studies between January 2012 and October 2014.

Selection criteria were the following:

- Referral of patient for staging of malignant disease by skeletal scintigraphy
- At least two SPECT/CT examinations with overlapping field of view (FOV)
- At least one osseous metastasis in SPECT/CT FOV with clear-cut and typical SPECT and CT abnormality
- Absence of any kind of metal or surgical implants in the FOV
- Access to measured prepared activity, time of measurement, injection time, residual syringe activity, time of residual syringe activity measurement, and patient weight

Overall, 19 patients conformed to the above criteria. As part of palliative therapy, 8 of 19 patients received bisphosphonates between both scans. The demographic and clinical data of these subjects are provided in Table 1; 16 patients were females suffering from metastatic breast cancer, while 3 were male with metastatic prostate cancer.

**Table 1:** Clinical data of all subjects included in the analysis

Subject	Type of cancer	Age	Time between scans (months)	Therapy between scans	Bisphosphonates
P001	Prostate	77	6	Leuprorelin/bicalutamide	None
P002	Breast	50	11	Tamoxifen	Zolendronate
P003	Breast	62	6	Tamoxifen	Zolendronate
P004	Breast	57	11	Denusomab/everolimus	none
P005	Breast	54	24	Tamoxifen	Zolendronate
P006	Breast	59	12	Fulvestrant	Zolendronate
P007	Breast	48	6	Denusomab	None
P008	Breast	71	6	Anastrazole	Zolendronate
P009	Breast	73	11	Exemastane/everolimus	None
P010	Breast	66	12	None	Zolendronate
P011	Breast	71	4	Denusomab/letrozole	None
P012	Breast	57	13	Anastrazole	None
P013	Breast	44	7	Letrozole	Zolendronate
P014	Prostate	75	15	Abiraterone/goserelin/docetaxelce	Zolendronate
P015	Breast	74	3	Denusomab/afinitor/exemstan	None
P016	Breast	42	12	Eribulin	None
P017	Breast	49	12	Letrozole	None
P018	Breast	71	16	Denosumab/trastuzumab/letrozole	None
P019	Prostate	78	14	Bicalutamide	None

### Data acquisition and reconstruction

In vivo patient imaging was acquired at our institution on either a Symbia T6 or a Symbia T2 (Siemens Molecular Imaging, Hoffman Estates, IL, USA). SPECT scans were carried out using low-energy high-resolution (LEHR) collimation, 256 by 256 matrix size, and a total of 120 projections (60 stops) over 360° with a dwell time of 15 s per stop. Following the SPECT acquisition, a low-dose CT scan was performed with 130 kV and 30 reference mAs using adaptive dose modulation (CARE Dose 4D). The CT data were generated with 5 and 3 mm slice thicknesses using smooth, medium, and sharp kernels (B08s, B41s, and B70s, respectively). The B08s kernel was solely used for SPECT attenuation correction.

In the study cohort, the injected activity was 8.0 ± 1.1 MBq (6.5 to 10.1 MBq) of ^99m^Tc-diphosphono-propanedicarboxylic acid (^99m^Tc-DPD) per kilogram of body weight for time point 1 and 7.6 ± 0.6 MBq (6.3 to 8.5 MBq) for time point 2, respectively (TP1/TP2). The mean total activity administered was 573.2 ± 105.4 MBq (330–750 MBq) and 542 ± 80.4 MBq (409–699 MBq), respectively. The slightly lower injected activity at TP 2 mainly reflects newer regulations on radioactive drugs in German law, as recommended by the “Bundesamt für Strahlenschutz”. The SPECT/CT scan was performed on average 231 ± 57 min (146–343 min) and 234 ± 37 min (186–303 min) after intravenous injection. The injected dose and time lapse between injection and acquisition for each subject, as well as patient weight, can be found in Table 2. The mean elapsed time between TP1 and TP2 was 10.3 ± 5 months, ranging from 3 to 24 months (see Table 1).

**Table 2:** Injected dose at time point 1 and 2 and time lapse between injection and acquisition for each patient scan

Subject	Weight (kg)	Injected dose TP 1 (MBq)	Injected dose TP 2 (MBq)	Time to acquisition TP 1 (min)	Time to acquisition TP2 (min)
P001	95	656	699	290	186
P002	65	583	500	249	248
P003	67	523	548	343	203
P004	88	625	614	237	292
P005	110	750	693	232	230
P006	62	463	468	301	228
P007	76	699	566	146	220
P008	71	515	587	290	280
P009	60	443	521	296	187
P010	60	608	499	252	234
P011	60	584	420	192	278
P012	65	614	475	163	287
P013	48	330	409	179	222
P014	90	708	620	172	211
P015	74	625	493	256	206
P016	66	450	534	252	303
P017	70	589	562	168	191
P108	76	493	493	173	218
P019	76	633	597	206	221

SPECT reconstruction was performed using Flash3D [13, 15, 16], an ordered subset expectation maximization (OSEM) reconstruction algorithm with depth-dependant 3D (axial and trans-axial) resolution recovery, scatter correction, and attenuation correction based on attenuation maps derived from the B08s CT data. Camera calibration and OSEM SPECT reconstruction parameters (four subsets, eight iterations, no post-smoothing) used for quantitative evaluations were carried out according to a previously published protocol [16].

### Data analysis

Both qualitative and quantitative image analyses were performed. Qualitative analyses were carried out on workstations using the Oncology and MM Basic tool of the Syngo.via software (Siemens Molecular Imaging, Hoffman Estates, IL, USA).

#### Qualitative evaluation

In the first step, the planar scintigrams and the SPECT/CT datasets from TP1 and TP2 were displayed and evaluated for metastatic lesions contained in a common FOV. As we generally acquired only one SPECT bed position, the FOV evaluated in this study was thus usually limited by that of the SPECT/CT. Of the lesions found, a maximum of six metastases detectable on both SPECT/CT scans of the patient in question were selected for visual and quantitative analysis. The selection criterion was a visually definable increase of uptake, with more focal metastases preferable. In total, 52 lesions from 19 patients were included. This process was performed by reader one only.

In the next step, the TP1 and TP2 image datasets were jointly displayed, and the uptake in the marker lesions was visually compared and classified as increased, decreased, or equal in uptake.

This analysis was performed independently from each other by two physicians for both planar scintigraphy and SPECT/CT. Reader 1 had 4 years’ experience in clinical nuclear medicine and 4 years’ experience in reading skeletal SPECT/CT. Reader 2 was board-certified with 28 years’ experience in clinical nuclear medicine and 9 years’ experience in reading skeletal SPECT/CT.

#### Quantitative evaluation

The delineation of the VOIs was performed using the standard clinical volumetric analysis tool provided by the camera’s vendor. The B70s CT data were used to drive the VOI selection in a fused SPECT/CT display. An ellipsoidal VOI was defined on the fused image representation around each lesion in question, and peak count values (average counts within a 1 cm^3^ sub-volume of interest about the VOI maximum) were measured. In the follow-up images, a VOI at an analogous anatomical position and of a comparable volume was drawn. Due to the manual fashion of the VOI definition, exact position and size of the VOI are subject to intra- and inter-reader variability.

When the lumbar or thoracic spine was included in the FOV, the spongeous bone of one vertebral body unaffected by SPECT or CT pathology both on the first and on the follow-up scan was selected as reference tissue. The mean count values of the reference tissues were measured by manually drawing VOIs in the inside of the vertebral body such that the borders coincided with the vertebral cortical bone shell, as reported previously [17]. Reference regions were available in 17 of 19 patients only. In these patients, a total of 46 lesions were available for quantitation. Of the two patients lacking a reference region, one had only the base of the skull included in the FOV, and the second showed a multifocal bone involvement in all vertebral bodies imaged with SPECT/CT.

Extracted count values of each VOI were converted to absolute activity concentrations according to previously mentioned methods [16]. All data were decay-corrected to time of injection in order to control for fluctuations in start time of the acquisition. Final values of quantitative tracer concentrations are thus defined at injection time.

After determining the local tracer activity concentration in kiloBecquerel per milliliter, normalization by injected activity (corrected for residual activity in the syringe) and body weight yielded SUVs. Additionally, the ratio between SUV_peak_ of the lesion and SUV_mean_ of the corresponding reference region was calculated to correct for global fluctuations in bone metabolism.

Similar to the Response Evaluation Criteria in Solid Tumors Version 1.1 (RECIST 1.1) criteria in CT and suggested positron emisson tomography response criteria in solid tumors version 1.0 (PERCIST criteria) in FDG-PET, a change of more than 25 % in tracer uptake was defined as significant [18–20]. The lesions were then again classified as progressive (>30 % increase), regressive (>30 % decrease), and stable (in between) [18–20]. This classification was performed on basis of the absolute quantification as well as the above described ratios.

Quantification of the target lesions and reference regions in SPECT/CT was carried out independently by the two readers.

### Statistical analysis

Inter- and intra-observer, as well as inter-method agreement were assessed using Cohen’s kappa, a statistical metric useful for measuring inter-rater agreement [21]. Cohen’s kappa $k$ is calculated on the basis of the observed agreement *p*_0_ between two raters and the random agreement *p*_e_ expected for statistically independent decisions of both raters: $k=\frac{p_0-{p}_e}{1-{p}_e}$. Landis and Koch provide guidance for interpreting values of $k$, which we consequently adapted for our evaluation and are displayed in Table 3 [22].

**Table 3:** Proposed scoring system of Landis and Koch (Biometrics 1977 [22]) for rating of the Kappa statistic

Kappa statistic	Strength of agreement
<0.00	Poor
0.00–0.20	Slight
0.21–0.40	Fair
0.41–0.60	Moderate
0.61–0.80	Substantial
0.81–1.00	Almost perfect

The variables were tested for differences using a Mann-Whitney *U* test. Correlations were tested using a two-sided Spearman’s rank correlation coefficient. Significance was accepted at *p* < 0.05.

In the “Results” section, we report quantitative measures, including tracer activity concentration expressed in SUV. The data are described by providing their mean values and their standard deviations (SD). Statistical analysis was performed using SPSS^©^ Version 19 (IBM, Copyright 2010, Armonk, New York, USA).

## Results

**Fig. 1:**
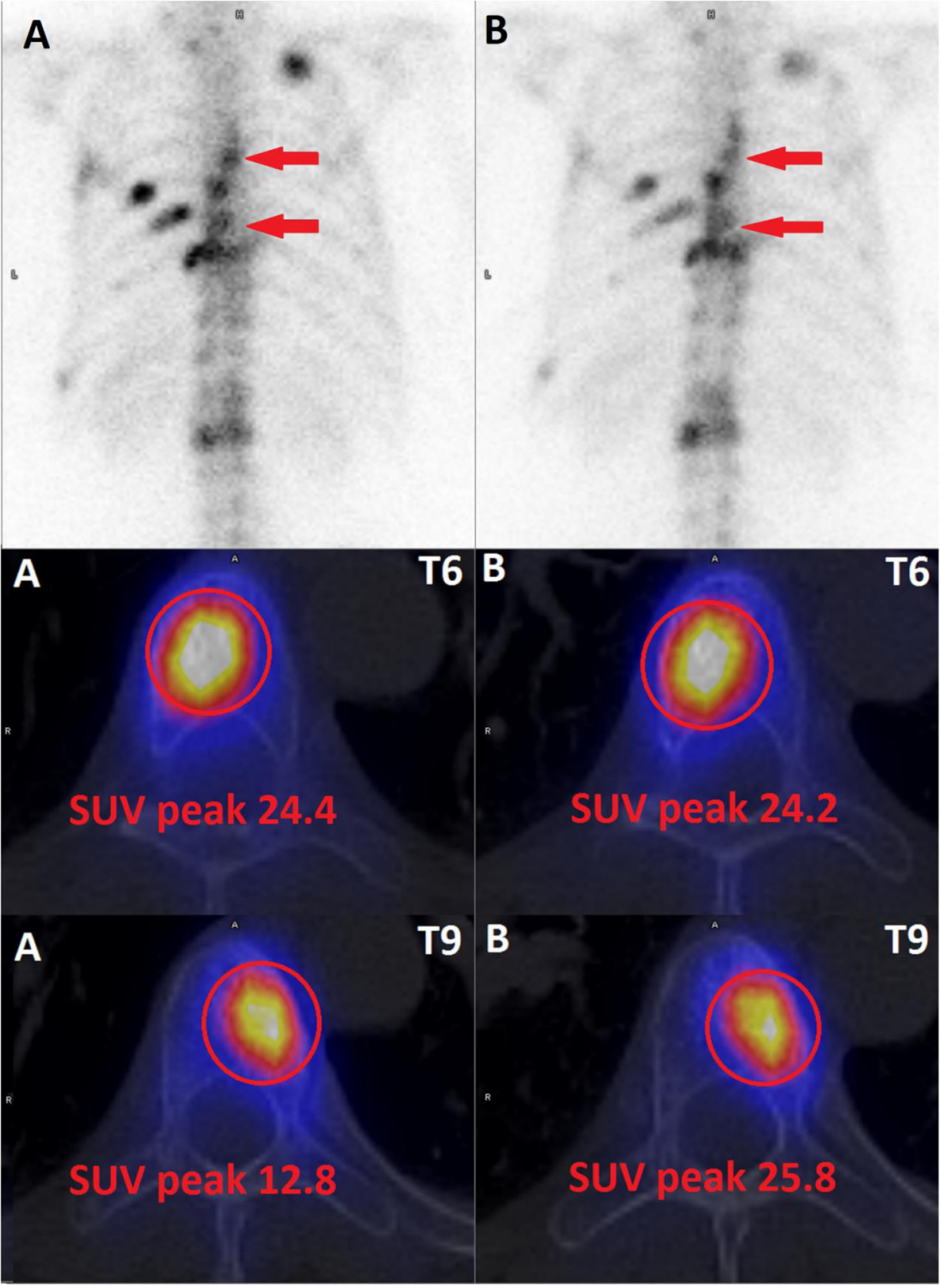
Planar bone scintigraphy and fused SPECT/CT at TP1 (**a**) and TP2 (**b**) of a patient with metastatic breast cancer. Both metastases in the thoracic spine were visually considered stable. Quantitative assessment revealed an increased tracer uptake for the lesion in thoracic vertebra body 9 (T9), whereas activity concentration of the lesion in thoracic vertebra body 6 (T6) was stable

Nineteen patients with a total of 52 bone lesions, characterized by both CT and bone scintigraphy as osseous metastases, were included in the quantitative analysis. The location of the analyzed lesions is listed in Table 4. A representative patient example is given in Fig. 1; 17 patients with in a total of 46 lesions were extracted for estimation of uptake ratios.

**Table 4:** Location of OMs and corresponding mean peak standard uptake values (SUV_peak_) with standard deviation (SD)

Region	Number	Mean SUV_peak_	SD SUV_peak_
Skull	2	17.1	9.5
Shoulder	3	11.1	7.7
Cervical spine	2	30.9	1.6
Thoracical spine	14	24.7	33.8
Lumbar spine	10	36.2	39.1
Pelvis	9	20.6	18.0
Ribs	12	19.5	13.3

### Qualitative evaluation

The respective numbers of classified lesions of the two readers using planar bone scintigraphies (BS) and SPECT/CT can be found in Tables 5 and 6. Inter-observer agreement of the visual analysis was *k* = 0.46 (*p* < 0.01) for planar scintigraphy and *k* = 0.35 (*p* < 0.01) for SPECT/CT (Table 7). The aggregated (both readers = 104 lesions) inter-method agreement between the visual analysis of planar scintigraphy and SPECT/CT was *k* = 0.36 (*p* < 0.01) (Table 8).

**Table 5:** Results from visual lesion classification compared to those from assessment of peak standard uptake value (SUV_peak_) in 52 metastatic bone lesions

	Progressive	Stable	Regressive	Discrepant lesions (*n*)
Reader 1 quantitative SPECT/CT	13	13	26	
Reader 1 planar BS	15	21	16	18
Reader 1 SPECT/CT	11	16	25	11
Reader 2 quantitative SPECT/CT	12	15	25	
Reader 2 planar BS	20	12	20	22
Reader 2 SPECT/CT	18	12	22	14

Lesions were classified either visually or based on the absolute peak tracer uptake. In this case, a change of more than 30 % in peak tracer uptake was rated as a significant increase (=progressive) or decrease (=regressive). All other lesions were classified stable. The listed number of discrepant lesions refers to the comparison of each visual analysis to the quantitative assessment

**Table 6:** Results from visual lesion classification compared to those from assessment of uptake ratios in 46 metastatic bone lesions

	Progressive	Stable	Regressive	Discrepant lesions (*n*)
Reader 1 uptake ratios SPECT/CT	12	20	14	
Reader 1 planar BS	14	18	14	14
Reader 1 SPECT/CT	10	15	21	16
Reader 2 uptake ratios SPECT/CT	10	22	14	
Reader 2 planar BS	19	9	18	23
Reader 2 SPECT/CT	18	8	20	19

Lesions (OM) were classified either visually or based on ratios of absolute peak tracer uptake in the OM divided by the mean tracer uptake in the reference region. In this case, a change of more than 30 % in the ratio was rated as a significant increase (=progressive) or decrease (=regressive). All other lesions were classified stable. The listed number of discrepant lesions refers to the comparison of each visual analysis to the quantitative assessment

**Table 7:** Inter-observer agreement as Kappa value for each method of classification

	Kappa
Visual planar	0.46
Visual SPECT/CT	0.35
Quantitative SPECT/CT	0.94
Uptake Ratio SPECT/CT	0.87

**Table 8:** Inter-method comparison of aggregated results for both readers (*n* = 2*52 = 104 lesions)

	Kappa
Visual planar vs. visual SPECT/CT	0.36
Visual planar vs. quantitative SPECT/CT	0.42
Visual planar vs. uptake ratio SPECT/CT	0.40
Visual SPECT/CT vs. quantitative SPECT/CT	0.62
Visual SPECT/CT vs. uptake ratio SPECT/CT	0.44
Quantitative SPECT/CT vs. uptake ratio SPECT/CT	0.55

### Quantitative evaluation

Across all lesions and both readers, average SUV_peak_ (±SD) of the osseus metastases (OMs) was 20.4 (±20.8) at TP1 and 16.4 (±11.5) at TP2. For the reference regions, average SUV_mean_ was 5.6 (±1.9) and 4.9 (±2.2) at TP1 and TP2, respectively. Both SUV measurements in lesions and background had a high correlation with *r* = 0.99 (*p* < 0.01) and *r* = 0.92 (*p* < 0.01), and the resulting classification showed an almost perfect inter-observer agreement with *k* = 0.94 (*p* < 0.01).

The ratio SUV_peak_ to SUV_mean_ was found to be 3.8 (±3.3) and 4.1 (±2.7) for TP1 and TP2, respectively, the resulting classification showed an inter-observer agreement of *k* = 0.87 (*p* < 0.01) (Table 7). In 30.4 % of the lesions, the classification assessed by uptake ratios was different compared to classification according to quantitative uptake alone (*k* = 0.55; *p* < 0.01) (Table 8).

### Comparison of qualitative and quantitative evaluation

Discrepancies between visual and quantitative assessment occurred in between 11 (21.1 %) and 22 (42.3 %) of the 52 lesions, depending on the reader and whether planar or SPECT/CT imaging was considered (Table 5).

The comparison of visual assessment and uptake-ratio analysis yielded discrepant findings in the same frequency (Table 6).

In Table 7, we report the according Kappa statistic for the inter-observer agreement.

When aggregating the classification results for both observers in order to compare inter-method agreement between quantitative and visual assessment of planar scintigraphy, *k* was 0.42 (*p* < 0.01). When comparing the same quantitative results to visual assessment of the SPECT/CT, *k* was 0.62 (*p* < 0.01). The results of the aggregated comparison of visual and quantitative assessment and the uptake ratios can be found in Table 8.

## Discussion

To the best of our knowledge, this is one of the first studies comparing visual and quantitative evaluation of disease progress in bone scans while reporting DPD uptake in healthy and diseased bone in absolute units and capitalizing on recent methodological advances in SPECT/CT (for a review, see Ritt et al. [14] and Bailey et al. [23]).

Studies on the efficacy of bone scans for therapy monitoring are scarce, but is has been shown that the results of bone scans and fluorine PET heavily influence therapy decisions in clinical practice: Hillner et al. investigated the influence of ^18^F-fluoride PET/CT on therapy management in 2217 patients with osseous metastases. In 40 % of the cases, a change in tumor therapy based solely on the results of the PET/CT was observed [7].

Despite the missing proof that progress as assessed by bone scintigraphy is correct, Imbriaco et al. introduced a method of measuring metastatic bone involvement in bone scintigraphy of prostate cancer—the so-called bone scan index (BSI) [24]—and could show that it features a high correlation with prostate-specific antigen (PSA) and low intra- and inter-observer variability. The BSI is calculated as the sum of the product of fractional bone weight and the percentage of tumor involvement for each bone in the body. However, primarily due to the time-intensive assessment process, the bone scan index has not become established in the clinical routine.

For other modalities, various semi-quantitative classification systems of tumor response in bony lesions have been previously researched.

In the late 1970s, the Union for International Cancer Control (UICC) and the World Health Organization (WHO) introduced a method in which criteria were based on conventional X-ray and planar skeletal scintigraphy [19, 25–27]. Dependent on tumor size development in X-ray imaging, lesions are classified as partially responsive (at least 50 % decrease), progressive (more than 25 % increase), and stable (in between). A disappearance of all lesions in skeletal scintigraphy is rated as complete remission.

The most commonly used MRI/CT tool for monitoring therapy is provided by the RECIST, first published in 2000 [19] and revised in 2009 [18]. By measuring certain target lesions, overall response is evaluated. Unfortunately, solitary bone lesions are characterized as immeasurable under this scheme, and only osteolytic lesions with involvement of surrounding soft tissue can be included.

### Quantitative uptake of osseous lesions

Average SUV_mean_ in morphologically healthy spongeous bone of the vertebra was 5.6 (±1.9) and 4.9 (±2.2) in our subjects, thus agreeing closely with data published earlier by our group for DPD uptake in the lower lumbar spine [17]. Malignant osseous lesions had a considerably higher uptake of ^99m^Tc-DPD than normal osseous tissue, with an average SUV_peak_ of 20.4 (±20.8) and 16.4 (±11.5) for TP1 and TP2, respectively.

Interestingly, osseous DPD uptake determined by SPECT was in the same range as that reported for ^18^F-fluoride measured by PET. In a study by Uchida et al., who investigated the influence of alendronate on bone metabolism in glucocorticoid-induced osteopenia, SUV_mean_ values are given as 5.9 in the lumbar spine of a bone-healthy control group [28]. Furthermore, Cook et al. reported maximum SUVs for ^18^F-fluoride in bone metastases from 11.4 to 98 (mean 37.2) in their group of five subjects with castrate-resistant prostate cancer undergoing a treatment with ^223^Ra-dichloride [29]. In our data, SUV_max_ rated 36.7 (±39.5) and 24.5 (±18.0) for TP1 and TP2, respectively.

The clinical impact of truly quantitative SPECT/CT has as yet not been established and is also beyond the scope of this current study, mainly due to our lack of a gold standard. Expectations are that quantitative workups could prove beneficial when diagnosing diseases affecting tracer uptake in a diffuse rather than focal manner such as osteoporosis [17] or three-vessel coronary artery disease [30]. Other potential benefits include improved personalized dosimetry of radionuclide therapies and further the use of SPECT/CT for monitoring therapy, e.g., in neoplastic disease, analogously to PET [23].

### Comparison of qualitative and quantitative evaluation

Based on our small dataset, we could show a large discrepancy between visual and quantitative analysis, yielding inconsistent results in up to 42.3 % of the inspected lesions. Furthermore, visual classification results from both planar scans and SPECT/CT images had only a fair to moderate inter-observer agreement with a $k$ of 0.46 and 0.35, respectively. One possible interpretation of this finding is that changes in tracer uptake between TP1 and TP2 were only minor, leading to differences in visual interpretation of the same scan [31]. In comparison, inter-observer agreement in quantitative evaluation was almost perfect with a *k* of 0.94.

Using the classification based on the uptake ratios, discrepancies to visual evaluation could be found in a comparable frequency, resulting in a correspondingly fair agreement. Whether uptake ratios or a quantitative evaluation are better suited for assessment of local tracer uptake changes could not directly be evaluated in this study, but agreement between both methods was substantial. However, inter-observer agreement using uptake ratios was slightly inferior compared to quantitation. This is most likely caused by the greater variability due to differing SUV_mean_ values between readers, which was effectively magnified by the smaller magnitude of the reported quantities when using uptake ratios.

Results on uptake-ratio analysis have been reported for fluoride PET. In 2011, Cook et al. published a study on therapy monitoring with ^18^F-fluoride-PET/CT in patients with castration-resistant prostate cancer treated with ^223^Ra-dichloride [29]. Five patients receiving 2 cycles of therapy 6 weeks apart underwent F-18-fluoride-PET/CT scans at baseline as well as in weeks 6 and 12 after institution of therapy. Although PSA showed a significant change in all patients, the scans were visually rated as unchanged. However, semi-quantitative analysis of tracer uptake revealed an agreement of changes in bone metabolism and the course of the PSA and serum alkaline phosphatase (ALP) values. Thus, though also limited in patient number, the authors could demonstrate the superiority of uptake ratios over visual assessment [29].

Based on our results and those reported in literature, there are strong indications that visual analysis of bone scans for longitudinal changes in tracer uptake of individual lesions is unreliable and should be substituted by semi-quantitative or quantitative evaluation.

### Limitations

As mentioned before, predictions about the clinical impact of such measurements cannot be made on the basis of our data due to the missing gold standard and our small patient number.

In particular, histological results are available only infrequently, and furthermore, tumor markers in certain tumor entities like breast cancer, which represents the majority of our study collective, are unreliable [32]. A further confounding variable is the flare phenomenon, which is caused by the temporary increase of reparative osteoplastic activity in metastatic bone lesions after treatment and occurs in prostate and breast cancer and can potentially lead to false-positive results [33]. In patients with breast cancer, this phenomenon was observed in up to 35 % of the cases in an average of 3.3 ± 1.4 months after therapy initiation. A stabilization of bone metabolism was seen within 6.2 ± 3.0 months [34]. Since, on average, 10.3 ± 5 months elapsed between our two scans, the influence of this phenomenon on our results should be only minor.

## Conclusions

In this study, we compared agreement of the visual evaluation of both planar scintigraphy and SPECT/CT with the quantitation of local tracer uptake in longitudinal bone scans of patients with osseus metastases. Compared to measuring DPD uptake in absolute units, visual evaluation of skeletal scintigraphies for change in tumor metabolism yielded inconsistent results in up to 42 % of the cases. Furthermore, visual analysis showed only moderate inter-observer agreement, while inter-observer agreement in quantitative evaluation was almost perfect. This suggests that longitudinal analysis of bone scans for changes in tracer uptake of individual lesions should be performed using quantitation of tracer uptake rather than solely by visual assessment to ensure that consistency in patient management is maximized.

## Abbreviations

^99m^Tc-DPD, ^99m^Tc-diphosphono-propanedicarboxylic acid; BS, bone scintigraphy; BSI, bone scan index; CT, X-ray computed tomography; FOV, field of view; MRI, magnetic resonance imaging; OMs, osseus metastases; PERCIST, positron emisson tomography response criteria in solid tumors version 1.0; PET, positron emission tomography; PSA, prostate-specific antigen; RECIST 1.1, Response Evaluation Criteria in Solid Tumors Version 1.1; SPECT, single photon emission computed tomography; SUV, standardized uptake value; TP, time point; VOI, volume of interest
